# Reimagining Dementia Care: A Complex Intervention Systematic Review on Optimising Social Prescribing (SP) for Carers of People Living With Dementia (PLWD) in the United Kingdom

**DOI:** 10.1111/hex.70286

**Published:** 2025-05-10

**Authors:** Jessica Marshall, Evie Papavasiliou, Louise Allan, Katherine Bradbury, Chris Fox, Matthew Hawkes, Anne Irvine, Esme Moniz‐Cook, Aimee Pick, Marie Polley, Amy Rathbone, Joanne Reeve, Dame Louise Robinson, George Rook, Euan Sadler, Emma Wolverson, Sarah Walker, Jane Cross, the SPLENDID Collaboration

**Affiliations:** ^1^ University of East Anglia Norwich UK; ^2^ University of Leeds, Woodhouse Leeds UK; ^3^ University of Exeter Exeter UK; ^4^ University of Southampton Southampton UK; ^5^ University of West London London UK; ^6^ Newcastle University Newcastle upon Tyne UK; ^7^ Meaningful Measures Ltd Portishead UK

**Keywords:** carers, dementia care, people living with dementia, primary care, social prescribing

## Abstract

**Introduction:**

Carers of people living with dementia (PLWD) face a range of complex needs, including medical, emotional, social and practical challenges, often exacerbated by social isolation and barriers to accessing support. Social prescribing (SP) addresses these needs by increasing access to non‐clinical support and services. However, existing research lacks detailed descriptions of SP interventions for carers of PLWD, with limited understanding of the needs they target, the reasons for participation, their effectiveness and their potential to improve outcomes for carers of PLWD.

**Methods:**

A complex intervention systematic review of SP for carers of PLWD was undertaken using iterative logic modelling and reported following the Preferred Reporting Items for Systematic Review and Meta‐Analysis (PRISMA‐CI) extension statement and checklist. Six databases and grey literature were searched, supplemented by hand searching reference lists of included studies. Results were screened in a two‐step process, followed by data extraction. Gough's Weight of Evidence Framework was used to assess the risk of bias in the included studies.

**Results:**

Fifty‐two studies were included. Findings indicated SP for carers of PLWD in the United Kingdom is varied and operates in a largely uncoordinated process involving initiation by diverse stakeholders and institutions across multiple sectors. The classification of SP interventions for carers of PLWD is inconsistent, and participation is often opportunistic. Positive outcomes included improved carer mood, social connections, practical support, quality of life and better PLWD–carer relationships. However, negative outcomes were associated with intervention suitability, emotional impact, relevance and strained PLWD–carer relationships.

**Discussion:**

While the evidence suggests SP is a promising intervention for carers of PLWD, its long‐term impacts, challenges of tailoring prescriptions to carers' needs and overcoming logistical issues remain. Additionally, further research is required to evaluate long‐term impact, investigate specific mechanisms to tailor SP to specific carer needs and explore in greater detail the PLWD–carer relationship and its effects on SP uptake and maintenance.

**Patient and Public Contributions:**

A PPI advisory group was involved in the review, including providing insights into review questions, the logic model, findings and results. The group consisted of one person living with dementia and a caregiver.

## 1 Introduction

### 1.1 Background and Rationale

As the number of people living with dementia (PLWD) in the United Kingdom continues to rise [1], so too does the number of carers of PLWD navigating a range of complex needs, including medical, emotional, social and practical challenges, frequently exacerbated by social isolation and barriers to accessing support [2, 3]. Post‐diagnostic support (PDS) is essential for improving the quality of life for PLWD and carers of PLWD [4]. However, this support is often hindered by a lack of health system organisation and inequalities in its provision [4]. Addressing the PDS needs of carers of PLWD requires a comprehensive approach that incorporates medical, psychological and social support. Social prescribing (SP) is one such approach, addressing the non‐clinical needs of carers of PLWD through community‐based interventions typically provided by voluntary and community groups, such as support groups, arts and crafts activities, and physical activities [5, 6]. Evidence suggests SP can promote social engagement, reduce loneliness and enhance overall well‐being [5, 7, 8]. However, integrating SP into dementia care is challenging due to inconsistent service provision, a lack of implementation guidelines or referral processes, insufficient funding and limited stakeholder alignment on how SP should be instigated or hosted [9, 10, 11].

Therefore, a comprehensive systematic review (CISR) to identify, describe and explore how carers of PLWD engage with SP interventions is required to examine the mechanisms, processes and circumstances involved and inform future implementation strategies and improve dementia care outcomes. This paper is *Part 2* of a two‐part series that reports the mechanisms, processes and outcomes of SP for both PLWD (Part 1) *and* carers of PLWD (Part 2). The series' results were split due to the differing needs of the two groups and the heterogeneity and breadth of the evidence identified in the original review. This approach enables detailed exploration, interpretation and evaluation of the findings. *Part 1* of the two‐part series, reporting findings for PLWD, is reported elsewhere and is submitted ‘in press’ [12].

The overall review questions across both *Part 1* (PLWD) and *Part 2* (carers of PLWD) of the CISR were:

1. What SP interventions are currently available for PLWD and/or their carers in the United Kingdom?
2. To which PLWD and/or their carers are SP interventions being delivered?
3. What are the mechanisms (incl. services and agents) by which SP interventions for PLWD and/or their carers are being instigated?
4. What are the processes through which PLWD and/or their carers receive SP interventions?
5. For what reasons/circumstances do PLWD and/or their carers participate in SP interventions?
6. What are the effects of SP on (i) PLWD and/or their carers and (ii) dementia‐related healthcare, and how are these measured?

### 1.2 Operating Definition

Both *Part 1* (PLWD) and *Part 2* (carers of PLWD) of the CISR define SP as ‘a means for trusted individuals in clinical and community settings to identify non‐medical, health‐related social needs and connect individuals to non‐clinical supports and services within the community by co‐producing a social prescription’ [13 (p. 9)]. This definition emphasises two core components: (1) the connector, a trusted individual who provides holistic support and a personalised care plan, and (2) the co‐produced care plan, developed in equal partnership to address non‐medical health‐related needs.

## 2 Methods

The review protocol, which focused on both PLWD and carers of PLWD, was registered on the Prospective Register of Systematic Reviews (PROSPERO; CRD42023428625) on 16 June 2023. Detailed methods are described elsewhere [13], but a summary is provided below.

### 2.1 Data Sources

Multiple electronic databases (MEDLINE, EMBASE, PsycINFO, CINAHL, Scopus and Cochrane/CENTRAL), grey literature sources (EThOS and CORE) and reference lists of included studies were searched.

### 2.2 Inclusion and Exclusion Criteria

**Figure 1:**
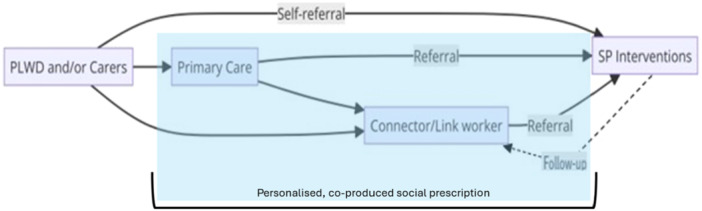
Social prescribing pathways illustrating the targeted literature scope for this review based on its working definition (highlighted in blue) [12, 14].

Inclusion and exclusion criteria were based on the PICOTS (Population, Intervention, Context, Outcomes, Timing, Setting) framework, targeting studies involving both PLWD and carers of PLWD who engaged with the core elements of the SP pathway in community settings (see Table S1 in Supporting Information). To address SP complexity and guide screening decisions, a figure representing possible pathways of SP, adapted from Husk et al. [14] in alignment with the working definition set for this review, was used (Figure 1) [12].

### 2.3 Search Strategy

The search strategy was developed with an academic librarian, who used relevant keywords and database‐specific terms for SP and dementia to tailor the search. A sample of the search string carried out in MEDLINE (Ovid) is provided in Supporting Information File S1. The search focused on UK‐based studies in English from 1 January 2003 to 15 June 2023 without methodological restrictions.

### 2.4 Study Selection

After de‐duplication in EndNote V.20, citations were imported into Rayyan for screening [15]. Two reviewers (J.M. and S.W.) independently assessed titles, abstracts and full texts, with disagreements resolved by a third reviewer (E.P.).

### 2.5 Data Extraction

Data relating to PLWD and carers of PLWD were extracted separately into a Microsoft Excel template designed to capture study characteristics and findings related to the review questions. Two authors (J.M. and S.W.) duplicated data extraction on 10% of studies to check accuracy, with adjustments made as necessary.

### 2.6 Quality Assessment

The Gough's Weight of Evidence (WoE) framework [16] was used to assess study quality. This framework was purposefully selected to appraise studies in this review due to its suitability for assessing diverse types of evidence, including qualitative, quantitative and mixed‐methods research. Given the complexity of SP interventions and the varied methodological approaches used in the included studies, a flexible and comprehensive appraisal tool was essential. The Gough (WoE) framework enables a structured evaluation of both methodological quality and relevance to the review questions, supporting a more nuanced synthesis of findings. Its use is well established in systematic reviews that draw on heterogeneous evidence, particularly within health and social care research.

The framework involves scoring studies across three domains: coherence (WoE A), design appropriateness (WoE B) and focus relevance (WoE C). An overall score (WoE D) was calculated for each study. One author (J.M.) performed the assessments, with 20% independently verified by another author (S.W.), which resulted in high agreement between the two reviewers. The quality assessment categorised studies as low, moderate or high quality. Typically, low‐quality studies would be excluded a priori; however, a deliberate decision was made not to exclude them from this review. This decision was justified because the existing evidence base specifically addressing SP interventions for carers is diffuse in nature. Excluding lower‐quality studies could potentially omit important contextual insights, novel approaches or innovative ideas that are essential for understanding the complexities and nuances of SP interventions. Furthermore, including all quality levels provides a comprehensive and transparent view of the current research landscape, clearly identifies methodological gaps and offers essential guidance for future research, policy development and practice improvements.

### 2.7 Data Synthesis

During narrative synthesis [17], PLWD data and carer of PLWD data were split and aligned to the review questions. Results were reported descriptively or thematically due to the heterogeneity of the data. This heterogeneity arose from the inclusion of multiple study designs, diverse intervention components, varied outcome measures and differing participant characteristics. Given these variations, a narrative approach allowed for an in‐depth descriptive and thematic interpretation of the data, capturing nuances and context‐specific insights effectively. The synthesis adhered to the AHRQ and PRISMA‐CI guidelines for complex interventions [18, 19]. Specifically, these guidelines provided a structured approach to systematically identify patterns and relationships within the data, ensuring methodological transparency and enhancing the interpretative rigour of the synthesis. Furthermore, narrative synthesis was particularly suitable for addressing research questions where numerical aggregation was not feasible or meaningful due to the varied methodological and conceptual frameworks across studies. It enabled a comprehensive and nuanced understanding of complex phenomena, which is critical in the context of SP interventions for carers of PLWD. This paper reports the data synthesis for carers of PLWD only; the synthesis for PLWD is reported elsewhere [12].

#### 2.7.1 Logic Modelling and PPI

Logic modelling was used throughout the review and was adapted iteratively [20] to map SP pathways, components and relationships at different stages of the review process [21]. During the final stage, the model was adapted and separated into two versions, one illustrating key components and relationships for PLWD [12] and the other for carers of PLWD. The final stage model for carers of PLWD is provided in Figure S1.

A Public and Patient Involvement (PPI) advisory group provided input regarding review questions, the logic model and emerging findings at different stages of the review process. The PPI group consisted of one person living with dementia and one carer with experience in caring for PLWD.

## 3 Results

**Figure 2:**
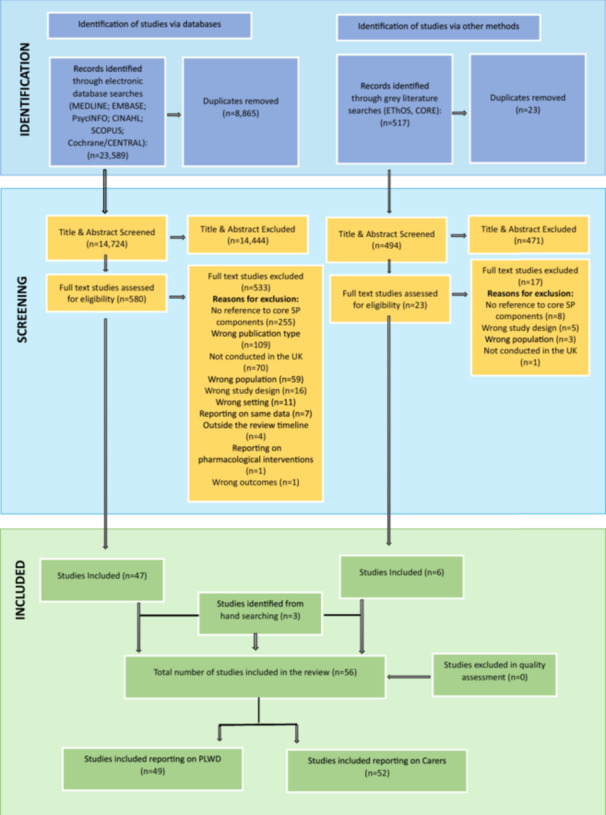
Preferred Reported Items for Systematic Reviews and Meta‐Analyses (PRISMA‐CI) flow chart.

The database searches retrieved 23,589 records related to PLWD and carers of PLWD. Following de‐duplication and title and abstract screening, 580 studies were assessed in full text for eligibility. 529 studies were excluded. Six studies were included from grey literature searches after deduplication and full‐text screening of the 517 studies identified. Three additional studies were included from manual searching of reference lists in the included studies. No studies were excluded during quality assessment. During data extraction and synthesis, the results were split into studies that reported data on PLWD and data on carers of PLWD; a total of 52 studies reported data on carers of PLWD and are included in this paper (see Figure 2 [12]). The data related to PLWD is reported elsewhere [12].

### 3.1 Characteristics of Included Studies

The 52 included studies on carers of PLWD comprise 41 original articles, 10 project reports and 1 PhD Thesis, using qualitative (*n* = 32), quantitative (*n* = 4), mixed‐methods (*n* = 12) and RCT (*n* = 4) designs. All were conducted in the United Kingdom: UK‐wide (*n* = 7), England (*n* = 35), Scotland (*n* = 5), Wales (*n* = 2), and England and Wales (*n* = 3), and published between 2005 and 2023. Table 1 summarises the included studies [4, 5, 7, 8, 11, 22, 23, 24, 25, 26, 27, 28, 29, 30, 31, 32, 33, 34, 35, 36, 37, 38, 39, 40, 41, 42, 43, 44, 45, 46, 47, 48, 49, 50, 51, 52, 53, 54, 55, 56, 57, 58, 59, 60, 61, 62, 63, 64, 65, 66, 67, 68].

**Table 1:** Characteristics of included studies.

Author(s)	Year	Aims	Participants	Setting	Design and analysis	Weight of evidence A‐B‐C/D
Database searches						
Ahmed et al. [22]	2018	To examine staff roles and tasks in Community Mental Health Teams (CMHT) and memory clinics.	Informants^a^	England	Quantitative; descriptive statistics	2.83–3–2.75/2.86
Akhtar et al. [23]	2017	To report on the recommendations from interviewed family carers of PLWD on their experiences of using dementia cafés.	Carers	England	Qualitative; thematic analysis	2.75–3–2.75/2.83
Al‐Janabi et al. [24]	2020	To determine the mechanisms by which health and care services affect family carers' well‐being.	Carers and informants	United Kingdom	Qualitative; thematic analysis	3–2.88–2.75/2.88
Atcha [25]	2018	To identify the socio‐cultural issues in accessing dementia services in the population living in Blackburn with Darwen in the Northwest of England.	PLWD; carers and informants	England	Qualitative; thematic analysis	3–2.88–2.63/2.84
Baker and Irving [26]	2016	To analyse the operation of a pilot social prescribing scheme established collaboratively between a Primary Care Trust (PCT) and Community Arts Organization (CAO), in the Northeast of England.	PLWD; carers and informants	England	Qualitative; thematic analysis	2.88–2.88–2.88/2.88
Bamford et al. [27]	2014	To test the transportability of a US case management model to primary care in England.	PLWD; carers and informants	England	Qualitative; thematic analysis	3–3–2.88/2.96
Bamford et al. [11]	2021	To identify the components of post‐diagnostic dementia support.	PLWD; carers and informants	England and Wales	Qualitative; thematic analysis	3–3–2.75/2.92
Bamford et al. [4]	2023	To develop an intervention to improve post‐diagnostic dementia care and support.	PLWD; carers and informants	England	Qualitative; framework analysis and realist evaluation	3–2.88–3/2.96
Brookes [28]	2017	To gather evidence to show whether Shared Lives could be a desirable service offer from the perspective of a carer or person with dementia and to support Shared Lives schemes to gain the confidence and skills they needed to be ‘dementia ready’.	Carers and informants	United Kingdom	Qualitative; thematic content analysis	2.96–2.88–2.75/2.86
Brooks et al. [29]	2014	To explore the impact of volunteering on experienced carers of people with dementia in a carer supporter programme (CSP).	Carers	England	Qualitative; thematic content analysis	2.96–2.88–2.75/2.86
Brown et al. [30]	2022	To explore family carers' experiences of the LBD Admiral Nurse service.	Carers	United Kingdom	Qualitative; narrative analysis	2.79–2.88–2.75/2.81
Burgess et al. [31]	2021	To explore the experience of people with dementia, family carers and occupational therapists taking part in the COTiD‐UK intervention.	PLWD; carers and informants	United Kingdom	Qualitative; thematic analysis	2.79–2.88–2.75/2.81
Charlesworth et al. [32]	2008	To determine whether a social support intervention (access to an employed befriending facilitator in addition to usual care) is effective compared with usual care alone, document direct and indirect costs, and establish incremental cost‐effectiveness.	Carers	England	Randomised controlled trial	3–3–2.88/2.96
Clarke et al. [33]	2013	To evaluate the Peer Support Networks and Dementia Advisors in the implementation of the National Dementia Strategy.	PLWD; carers and informants	England	Mixed‐methods; descriptive and inferential statistics; thematic analysis	2.92–3–2.88/2.93
Clarke et al. [34]	2018	To identify ways in which Dementia Advisors (DAs) and Peer Support Networks (PSNs) contribute to the well‐being and resilience of people with dementia and care partners.	PLWD; carers and informants	England	Mixed‐methods; descriptive and inferential statistics; content analysis	3–3–2.88/2.96
Egdell [35]	2012	To examine the factors that shape experiences of the development of support networks in informal dementia care: the difficulties in diagnosing dementia; the distinctions between social, support and care networks; caregivers' access to resources; and the expectations of the caregiver role.	Carers	England	Qualitative; thematic analysis	2.92–3–2.88/2.92
Egdell [36]	2012	To highlight the issues that health and social care practitioners need to take into account so that they can appropriately provide and target support for carers of people with dementia.	Carers	England	Qualitative; thematic analysis	2.83–2.75–2.88/2.82
Femiola and Tilki [7]	2017	To understand the challenges faced by people with dementia and their carers and what they felt they needed for the Dementia Peer Support Project.	PLWD and carers	England	Qualitative; thematic analysis	2.83–2.75–2.75/2.78
Field et al. [37]	2019	To examine the acceptability of the intervention for participants in the United Kingdom and to inform its adaptation, before a randomised controlled trial.	PLWD and carers	England	Qualitative; thematic analysis	3–3–2.75/2.92
Field et al. [38]	2021	To explore and examine influences on the uptake of psychosocial interventions by people with early dementia after diagnosis.	PLWD; carers and informants	England	Qualitative; thematic analysis	3–3–2.88/2.96
Giebel et al. [39]	2021	To explore the experiences of accessing post‐diagnostic dementia care for people living with dementia and carers both before and since the Covid‐19 pandemic, and potential associated inequalities.	PLWD and carers	England	Qualitative; thematic analysis	2.92–2.88–3/2.93
Giebel et al. [40]	2021	To evaluate a socially prescribed community service for PLWD and family carers.	PLWD and carers	England	Quantitative; descriptive and inferential statistics	3–2.88–2.88/2.92
Giebel et al. [41]	2021	To explore potential health inequalities influencing care pathways for people living with dementia and their family carers.	PLWD and carers	England	Qualitative; thematic analysis	3–3–2.75/2.92
Górska et al. [42]	2016	To evaluate the impact of the pilot FGC service, delivered to people with dementia and their families, in terms of the experience of care provision by families and care professionals involved in the project.	Carers and informants	Scotland	Qualitative; thematic content analysis	3–3–2.75/2.92
Greenwood et al. [43]	2017	To investigate in‐depth informal carers' experiences of attending cafés.	Carers	England	Qualitative; thematic analysis	3–3–2.88/2.96
Griffiths et al. [44]	2021	To understand the experiences of individuals with dementia or caring for someone with dementia, before and after a 12‐week relational counselling intervention.	PLWD and carers	United Kingdom	Qualitative; framework analysis	3–3–2.88/2.96
Griffiths et al. [45]	2022	To generate initial prospective theory building to develop a Dementia Support Worker intervention for PLWD and carers.	PLWD; carers and informants	United Kingdom	Qualitative; thematic analysis and realist evaluation	2.92–3–3/2.96
Hewitt et al. [46]	2013	The aim of this preliminary project was to identify possible benefits of a structured group gardening programme for people with YOD.	PLWD and carers	England	Mixed‐methods; descriptive and inferential statistics; thematic analysis	2.92–3–2.75/2.89
Hoskins et al. [47]	2005	To evaluate the effectiveness of interventions provided by a Community Mental Health Team (CMHT) in reducing stress in carers of individuals with dementia.	PLWD and carers	Wales	Quantitative; descriptive and inferential statistics	2.92–3–3/2.97
Kelly and Innes [48]	2016	Reports the views of the project held by people newly diagnosed with dementia and their family members; as such it builds on the body of literature focusing on the views of people with dementia and their carers.	PLWD and carers	Scotland	Qualitative; thematic analysis	3–3–2.88/2.96
Killin et al. [49]	2018	To determine the feasibility of improving the quality of life of people with dementia (PWD) and their families with the DSP by adopting a qualitative approach, focusing on the needs of families recently diagnosed with dementia, the work they do to address these needs and how the DSP may have been used to this end.	PLWD and carers	Scotland	Qualitative; framework analysis	3–3–2.75/2.92
Levin et al. [50]	2018	To examine three interpretations of post‐diagnostic support (PDS) for dementia, to understand how best to support people recently diagnosed with dementia.	Informants	Scotland	Mixed‐methods; descriptive and inferential statistics; thematic framework analysis	2.96–2.88–2.75/2.86
Ling et al. [51]	2023	To explore the effect of providing ongoing support to people recently diagnosed with dementia and their carers.	PLWD; carers and informants	England	Qualitative; thematic analysis	2.96–3–2.88/2.95
Mac Rae et al. [52]	2022	To generate new evidence on the social impact of Dementia Friendly Walking Football (DFWF) that would inform the development of this activity within society and provide feasibility data to inform a future more extensive research study.	PLWD; carers and informants	Scotland	Qualitative; thematic analysis	3–3–2.88/2.96
Maio et al. [53]	2016	To assess the effectiveness of the Admiral Nurses' approach from the perspective of family carers who had accessed their service to provide information for continuous improvement of practice, as well as providing evidence of users' satisfaction and effectiveness for commissioning purposes.	Carers	England	Quantitative; descriptive and inferential statistics	2.92–2.88–2.88/2.89
McDonald and Heath [54]	2008	To explore the provision of services for people with dementia and their carers in the three counties of Norfolk, Suffolk and Cambridgeshire in the area of the former Eastern Strategic Health Authority.	Carers and informants	England	Qualitative; thematic analysis	2.83–2.88–2.75/2.82
Milne et al. [55]	2014	To evaluate programme results and effectiveness of a course for carers of newly diagnosed PLWD.	Carers	England	Mixed‐methods; descriptive and inferential statistics; content analysis	2.96–3–2.88/2.95
Mountain et al.^b^ [56]	2022	To determine the clinical effectiveness and cost‐effectiveness of an intervention to promote self‐management, independence and self‐efficacy in people with early‐stage dementia.	PLWD and carers	England	Randomised controlled trial	3–3–3/3
Piercy et al. [57]	2018	To report the evaluation of an integrated service, introduced as part of a local health and social care strategy to improve post‐diagnostic dementia care.	PLWD; carers and informants	England	Mixed‐methods; descriptive statistics and framework analysis	3–3–2.88/2.96
Prendergast et al. [58]	2023	To conduct interviews with stakeholders of a Shared Lives (SL) day support service to explore mechanisms and outcomes for the service.	PLWD; carers and informants	Wales	Qualitative; framework analysis	3–3–2.88/2.96
Sprange et al. [59]	2021	To identify the barriers and facilitators to the implementation of a complex psychosocial intervention though a study exploring the experiences of participants, carers and interventionists during a trial.	PLWD; carers and informants	England	Qualitative; framework analysis	2.92–3–3/2.97
Wenborn et al. [60]	2021	To estimate the clinical effectiveness of Community Occupational Therapy for people with dementia and family carers–UK version (Community Occupational Therapy in Dementia–UK version [COTiD‐UK]) relative to treatment as usual (TAU).	PLWD and carers	United Kingdom	Randomised controlled trial	3–3–3/3
Wheatley et al. [61]	2021	To examine common barriers to the delivery of PDS for dementia in England and Wales including services from all sectors. We additionally describe a range of practical solutions used successfully by providers to address common barriers.	PLWD; carers and informants	England and Wales	Qualitative; thematic analysis	2.96–3–2.75/2.90
Wheeler et al. [62]	2015	To evaluate the Citizen Advice Bureau service provision, effectiveness and usefulness for service users.	PLWD; carers and informants	England	Mixed‐methods; descriptive statistics and thematic analysis	2.92–2.75–2.88/2.85
Willis et al. [63]	2009	To complete a qualitative investigation into the satisfaction with the service of those assessed and treated using the Croydon Memory Service Model (CMSM).	PLWD and carers	England	Qualitative; content analysis	2.96–3–2.88/2.95
Woods et al. [64]	2012	To assess the effectiveness and cost‐effectiveness of joint reminiscence groups for people with dementia and their family caregivers as compared with usual care.	PLWD and carers	England and Wales	Randomised controlled trial	3–3–3/3
Grey literature						
Ahmed et al. [65]	2017	To improve access to dementia services for BME communities in Salford increase carer identification and registration raise awareness of the needs of Salford's diverse communities; and to increase staff knowledge/develop evidence‐based decision‐making relating to minority communities who may access dementia services/general health and social care‐related services in Salford.	Informants	England	Qualitative; thematic analysis	2.83–2.75–2.75/2.78
Dayson et al. [66]	2014	To assess the impact of the pilot for its key stakeholders; to assess whether the aims and outcomes of the project had been achieved; to provide analysis of costs–benefits and return on investment, including assessing the cost savings and efficiencies to the NHS; to assess the effectiveness of the service delivery model; to establish a business case for future funding	Service Users (incl. carers of PLWD)	England	Mixed‐methods; descriptive and inferential statistics; thematic analysis	2.83–2.88–2.75/2.82
Dayson et al. [67]	2016	To evaluate the social and economic impact of the Rotherham Social Prescribing Service for people with long‐term health conditions	Service Users (incl. carers of PLWD)	England	Mixed‐methods; descriptive and inferential statistics; thematic analysis	2.88–2.75–2.75/2.79
Dayson et al. [8]	2020	To assess the effectiveness of the Rotherham Social Prescribing Service in improving health and social care outcomes for individuals with long‐term health conditions	Service users (incl. carers of PLWD)	England	Mixed‐methods; descriptive and inferential statistics; thematic analysis	2.79–2.75–2.75/2.76
Goodman et al. [68]	2019	To identify whether dementia‐friendly communities (DFCs) support people living with dementia and their carers to maintain their independence and feel valued members of their local community and, if so, which approaches have worked best and at what cost for which groups of people.	PLWD; carers and informants	England	Mixed‐methods; descriptive and inferential statistics; thematic analysis	2.88–2.75–2.75/2.79
Palmer et al. [5]	2017	To evaluate the benefits and limitations of a social prescribing pilot which took place in the Clocktower locality (London Borough of Bexley) over a 24‐month period, and this study forms the main body of the study.	Service users (Incl. PLWD and carers)	England	Mixed‐methods; descriptive and inferential statistics; thematic and narrative analyses	2.75–2.75–2.75/2.75

^a^ Individuals who provide supplementary or corroborative information about others or situations based on their observations, interactions and knowledge, offering insights and data from their own perspective, thereby providing a broader understanding and context about the primary subjects.

^b^ Data from the qualitative study embedded in the randomised controlled trial, as detailed in the report, were extracted from the paper by Sprange et al. [59], which focused solely on qualitative findings and preceded the report by Mountain et al. [56].

### 3.2 Heterogeneity, Focus and Nature of Available Evidence

The 52 included studies noted significant heterogeneity in study design, including carer demographics, SP intervention types, comparison groups and outcome measures.

The studies varied in focus and the type of evidence reported, particularly:

1. Reporting of the SP pathway: Reflecting the complexity of SP, most studies did not report all pathway elements. However, studies were included if they addressed core components (connector, personalised care plan, and engagement or reference to a non‐clinical service or activity) as referenced in the internationally accepted definition of SP [69].
2. Nature of evidence: Some studies relied on informants providing insights on carers of PLWD or generalised findings across various services or populations that included carers of PLWD.

Given the diversity of intervention approaches, outcomes measured and populations studied, conducting a quantitative synthesis was not feasible. Specifically, methodological heterogeneity (e.g., variations in study designs and outcome measurements), clinical heterogeneity (e.g., diverse population characteristics, intervention types and comparator conditions) and a general lack of standardised data necessary for calculating standardised effect sizes posed significant barriers. Attempting a meta‐analysis under these conditions could have risked under‐representing or misrepresenting the evidence base. Thus, the chosen narrative synthesis approach best accommodates the broad research questions and the diffuse nature of available evidence and ensures a comprehensive representation and meaningful interpretation of existing data.

Due to this heterogeneity, results are presented narratively, descriptively or thematically depending on available evidence. A concise summary of the results is visually represented in the final iteration of the logic model included in this systematic review (see Figure S1 in Supporting Information). Reporting follows the structure of the model, that is, participants, interventions/services, mechanisms, processes, reasons/circumstances and outcomes.

### 3.3 Participants

Participants refer to carers of PLWD or informants of carers of PLWD. Gender information was reported in 26 of the included studies, with 22 studies reporting exclusively [25, 58] or predominantly [4, 11, 23, 29, 30, 35, 36, 38, 39, 40, 41, 42, 43, 44, 47, 53, 59, 60, 63, 64] on female participants. Age was reported in 24 of the included studies, with most (*n* = 14) reporting ages between 40 and 90 years [11, 23, 29, 30, 35, 36, 39, 41, 43, 47, 48, 55, 56, 59]. The relationship of the carer to the PLWD was reported in 25 of included studies [23, 24, 25, 29, 30, 31, 32, 35, 36, 37, 38, 39, 40, 41, 43, 44, 47, 48, 49, 52, 55, 56, 59, 63, 64]: spouses (*n* = 24), son or daughter (*n* = 21), grandchild (*n* = 2), friend (*n* = 7), sibling (*n* = 7), daughter or son in law (*n* = 4), niece/nephew (*n* = 2), cousin (*n* = 2), neighbours (*n* = 1) and other (*n* = 3). Finally, eight studies reported living arrangements [31, 32, 36, 38, 39, 53, 55, 56], with most carers cohabiting with the PLWD they care for [36, 38, 39, 53, 55, 56].

### 3.4 Interventions/Services

46 studies reported specific kinds of SP interventions for carers of PLWD or were delivered via the carer to help support both the PLWD and the carer of the person living with dementia [4, 5, 7, 8, 11, 23, 25, 26, 29, 30, 31, 32, 33, 34, 35, 36, 37, 38, 39, 40, 41, 42, 43, 44, 45, 46, 47, 48, 49, 50, 51, 52, 53, 54, 55, 56, 57, 58, 59, 60, 61, 62, 63, 64, 66, 67]. These were predominantly umbrella interventions, which comprise a range of activities and services, including: *cognitive interventions* to support carers to support PLWD, such as memory clinics and reminiscence [35, 41, 63, 64]; *case management interventions*, including PDS, signposting, dementia advisor services, financial welfare, advice and advocacy, Admiral Nurses, and clinical advice and information [5, 11, 30, 33, 34, 48, 50, 53, 57, 62]; *psychosocial interventions* like dementia cafes, support groups, community engagement group, befriending and peer support networks [4, 5, 7, 11, 23, 25, 29, 30, 32, 33, 34, 35, 36, 38, 39, 41, 43, 45, 47, 50, 52, 54, 55, 56, 58, 59, 67]; *physical well‐being interventions*, such as exercise classes and leisure centre access [40]; *counselling interventions*, including carer counselling, relational counselling and family counselling [8, 42, 44]; *arts‐based and creative interventions*, including art, gardening and cooking [26, 41, 46]*; occupational therapy interventions* [31, 37, 60]; and *digital interventions*, such as a digital support platform [49].

Within these umbrella interventions, activities varied considerably, as did the intervention components (e.g., individualised support or group sessions), frequency or duration of the sessions, and mode of delivery (in person, online, telephone or email), therefore, highlighting the variability of SP interventions and services for carers of PLWD.

### 3.5 Mechanisms and Processes

Across the studies, the connector role manifested at different points in the SP process. In 38 studies, the referrer instigated the SP process and referred the carer of the person living with dementia to a connector that was part of a separate organisation from the referrer [4, 5, 7, 8, 11, 22, 25, 26, 28, 29, 30, 31, 33, 34, 36, 37, 38, 39, 40, 42, 45, 47, 48, 49, 54, 55, 56, 57, 58, 59, 60, 61, 62, 63, 64, 66, 67, 68]. However, in 6 studies, the referrer embodied the connector role [23, 35, 44, 46, 51, 65], and directly signposted, through the use of a personalised care plan, carers of PLWD to SP interventions, and in 8 studies the connector was reported as the sole instigator (no referrer) of the SP process for carers of PLWD [24, 27, 32, 41, 43, 50, 52, 53].

In 44 studies, the SP process began and was instigated by a *referral* [4, 5, 7, 8, 11, 22, 23, 25, 26, 28, 29, 30, 31, 33, 34, 35, 36, 37, 38, 39, 40, 42, 44, 45, 46, 47, 48, 49, 51, 54, 55, 56, 57, 58, 59, 60, 61, 62, 63, 64, 65, 66, 67, 68]. These included: referrals from *primary care* settings such as GPs, Admiral Nurses, dementia navigators, mental health nurses, clinical psychologists, occupational therapists and old‐age psychiatrists [4, 5, 7, 11, 22, 25, 26, 33, 34, 35, 36, 37, 40, 42, 44, 45, 47, 49, 51, 55, 56, 59, 61, 62, 63, 65]; *secondary care* services such as clinicians from memory services, memory clinics and community mental health services [5, 7, 8, 11, 23, 26, 28, 31, 33, 37, 38, 39, 40, 44, 46, 49, 51, 54, 56, 59, 60, 64, 66]; and *charities and voluntary sector organisations* including Alzheimer's Society, Age UK and Alzheimer's Scotland [5, 7, 8, 26, 29, 30, 33, 35, 36, 38, 39, 44, 46, 49, 51, 54, 56, 59, 60, 64, 66]. The SP process was also instigated by *self‐referrals* [7, 33, 35, 49, 51, 56, 57, 58, 59, 60, 62, 64] or *family referrals* [7, 47, 51, 57], highlighting the diverse number of stakeholders and networks spanning the healthcare system involved in instigating the SP process.

Studies reported a diverse range of individuals acting as connectors for carers of PLWD; however, inconsistency of the terms used to describe connectors was evident across all included studies, and in some cases, it was difficult to establish whether the connector was addressing the needs of the carer or the person living with dementia. Examples of individuals who were acting as connectors in studies reporting carer of PLWD data included: *clinical staff* such as clinical dementia leads, Admiral Nurses, link workers and well‐being practitioners [4, 5, 8, 23, 26, 30, 33, 34, 39, 40, 41, 43, 44, 45, 46, 50, 51, 53, 57, 61, 65, 66, 67]; staff from *memory clinics* and *mental health teams* [22, 25, 55, 61, 63]; *multidisciplinary teams* comprising of staff from different specialties such as occupational health or psychology [24, 27, 31, 36, 37, 38, 41, 47, 51, 61]; and personnel from the *third sector* or *voluntary and community‐based organisations and enterprises*, including befrienders, peer supporters and dementia advisors [7, 11, 28, 29, 32, 35, 42, 44, 46, 48, 49, 51, 52, 54, 57, 58, 61, 62, 68]. It is important to note that research teams, particularly in studies that assessed the effectiveness of SP, played a significant role in facilitating these connections post‐referral [56, 59, 60, 64]. Furthermore, terms relating to connectors were used interchangeably across sectors, and in some cases, roles and the connectors' place in the SP process were not explicit.

Thirty‐two studies reported the mechanisms of delivery in SP interventions and services for PLWD carers [4, 7, 23, 24, 26, 29, 30, 31, 33, 34, 36, 37, 40, 42, 43, 44, 47, 48, 49, 50, 51, 52, 53, 54, 55, 56, 58, 60, 61, 62, 63, 64]. These diverse mechanisms comprised *clinical staff* and *specialised therapists* [4, 24, 31, 37, 44, 47, 48, 55, 56, 60, 63, 64] and *non‐clinical staff* and *staff from voluntary and community sector enterprises* and *charities* [7, 23, 26, 29, 30, 33, 34, 36, 40, 42, 43, 49, 50, 51, 52, 53, 54, 58, 61, 62]. And finally, 37 studies reported the mechanism of the overarching organisation(s) that provided or commissioned the SP services or interventions for PLWD carers [4, 5, 11, 24, 26, 29, 30, 31, 32, 33, 34, 35, 36, 37, 38, 40, 41, 42, 43, 44, 47, 48, 49, 50, 51, 52, 53, 54, 55, 56, 57, 60, 61, 62, 63, 64, 67]. These included: *voluntary and community sector enterprises* and *charities*, such as Age UK, Alzheimer Scotland and faith‐based organisations [4, 5, 11, 24, 29, 30, 32, 33, 34, 35, 36, 37, 38, 40, 41, 43, 44, 47, 51, 52, 53, 54, 61, 67]; *public sector organisations*, such as NHS services and local authorities [11, 26, 31, 33, 34, 37, 38, 40, 41, 42, 47, 50, 53, 55, 56, 60, 61, 63, 64]; *integrated services*, which featured collaborations between healthcare service, charities, local government, community service and/or academic institutions [48, 49, 57, 62].

### 3.6 Reasons/Circumstances

Of the 52 included studies, 32 reported reasons for (motivations) and/or against (barriers) participating in SP interventions for carers of PLWD [4, 7, 8, 11, 23, 24, 25, 26, 29, 30, 31, 32, 33, 34, 35, 36, 37, 38, 39, 40, 41, 42, 43, 44, 45, 46, 47, 48, 55, 57, 58, 66] (see Table 2). The primary reasons for participating in SP interventions included seeking *emotional support*, such as peer support, creation of a supportive space and emotional gains [11, 23, 26, 29, 33, 34, 36, 43, 45, 55]; *practical support*, including signposting, financial advice, insight into dementia and action planning [4, 30, 31, 33, 37, 41, 42, 48, 55, 57, 58, 66]; *social and community engagement*, such as socialisation, educating the community, engaging in meaningful activities and time away from caring [8, 23, 29, 33, 35, 36, 40, 43, 55]; *increasing knowledge and/or empowerment*, including increasing confidence, self‐esteem and dementia knowledge [7, 29, 30, 31, 34, 38, 42, 45, 55]; and *coping strategies* [37, 44, 45].

**Table 2:** Reasons for (motivations) and against (barriers) carers of PLWD participating in SP interventions.

	Theme	Sub‐themes for carers
Reasons FOR	Emotional support	Emotional gains (connection, belonging, identity, confidence, pride and self‐esteem) Speaking frankly about difficulties Relief from caregiving responsibilities
	Practical support	Practical information (power of attorney and financial aid) Reliable support Single contact point Structured sessions
	Social and community	Socialisation with peers Increasing social networks Shared experiences Community education
	Adjustment and coping	Coping with caregiving challenges Managing negative changes Developing coping mechanisms Willingness to try new activities
	Knowledge and empowerment	Knowledge improvement (dementia and caregiving strategies) Realistic goals Empowerment through education—specialist knowledge (Admiral Nurse)
	Trust and reliability	Reliable support from empathetic experts Supportive atmosphere Dependability of professionals (e.g., Admiral Nurse) Open referral system
	Activity engagement	Enjoyment of shared activities Structured and meaningful activities Volunteering support Socialising separately from PLWD
	Shared knowledge and experience	Sharing knowledge about dementia Peer support Gaining insight from others' experiences Group friendships
Reasons AGAINST	Lack of cultural sensitivity	Services lack cultural sensitivity Lack of provision for marginalised populations/ethnic minorities
	Overwhelming information	Overwhelmed by advice and information Poor communication between sectors
	Transport issues	Lack of transport Transport difficulties (poor public transport) Geographic inequities
	Health and physical barriers	Carer health issues Unable to attend with PLWD due to ill health
	Access and awareness	Lack of information about available support Insufficient information post‐diagnosis Hard to access information
	Timing and adjustment	Need time to adjust to the diagnosis Intervention offered too soon Too busy with other responsibilities
	Inappropriate activities	Activities not appropriate for YOD (young‐onset dementia)
	Financial concerns	Cost of interventions Transport costs
	Psychosocial issues	Anxiety, stress, depression and a sense of guilt Not wanting to burden others with problems
	Communication issues	Poor communication between facilitators and carers Lack of referrals from primary care to social prescribing
	Dependence and autonomy	Carers feel the onus is on them to locate support Carers' assumptions that they can cope alone
	Family dynamics	Including PLWD in decision‐making can be distressing
	Professional support and trust	Dubious about intervention and not knowing the occupational therapy Lack of knowledge by healthcare professionals
	Social isolation and peer issues	Gender imbalance in groups Feeling isolated
	Practical barriers	Practical help over signposting
	Resource limitations	Long waiting lists and limited availability Postcode lottery of services Insufficient resources
	Emotional burden	Anxiety from seeing others further in the dementia journey

Significant barriers preventing carers of PLWD from participating in SP interventions included: *a lack of cultural sensitivity*, where services were not tailored to specific socio‐cultural needs [11, 25]; *practical barriers*, in particular a lack of transport options to SP services, financial costs linked to attendance, lack of local SP resources and inability to access a link worker [7, 11, 30, 31, 32, 33, 35, 36, 38, 39, 40, 41, 45, 48]; *health and physical barriers*, including physical impairments and carer ill health [36, 38, 41, 45]; *psychosocial and emotional issues*, such as feeling like a burden to the health system, anxiety about attending group settings and seeing others further along in the dementia journey, and SP not considering the carer's emotional or psychosocial needs [4, 11, 29, 35, 36, 37, 42, 43, 44, 45, 48]; and finally, *information* and/or *communication issues*, such as inconsistent communication between services, a disjointed referral, a lack of information about SP, SP information too overwhelming, and poor matching of services to carer needs [24, 31, 35, 38, 39, 42, 46, 48, 58]. These themes emphasise the need for more accessible, culturally sensitive, informed and co‐ordinated approaches to SP support for carers of PLWD.

### 3.7 Outcomes

Positive and negative outcomes from carers of PLWD participating in SP interventions were identified in 44 studies [5, 7, 8, 11, 23, 24, 25, 26, 27, 29, 30, 31, 32, 33, 34, 35, 36, 37, 38, 39, 40, 41, 42, 43, 44, 45, 46, 47, 48, 49, 51, 52, 53, 54, 55, 56, 57, 58, 60, 61, 62, 63, 64, 68] (see Table 3).

**Table 3:** Positive and negative outcomes for carers of PLWD from participation in SP interventions.

	Theme	Sub‐themes for carers
Positive outcomes	Enhanced independence	Reduced caring load Respite from caregiving duties Empowerment through knowledge and support
	Improved mood and well‐being	Enhanced mood and hope for the future Reduced stress and anxiety Improved emotional responses
	Social connectedness	Increased socialisation and peer support Friendship and shared experiences with other carers Sense of belonging
	Mental and cognitive benefits	Better understanding of dementia Improved coping skills and patience Normalisation of the dementia experience
	Empowerment and identity	Retained sense of identity and purpose New carer identity and connection to other carers Feeling valued and supported
	Practical support and resources	Better decision‐making and planning Assistance with financial and legal matters Support from health services
	Quality of life improvements	Improved quality of life Enhanced well‐being and resilience Increased confidence in caregiving capabilities
	Positive relationships	Improved relationship with PLWD Reduced conflict in the care relationship Support from peers and health professionals
	Acceptance and adjustment	Facilitated acceptance of dementia diagnosis Better equipped to cope with caregiving Shifting perspectives and strategies
	Security and comfort	Feeling of security and relief Continuity of care and support Sense of comfort and reduced burden
Negative outcomes	Intervention suitability	Feeling poorly prepared due to a lack of information provision Tendency to rely on one‐size‐fits‐all approach Increased burden to facilitate emotional and practical support Carer/family burden in including PLWD in decision‐making
	Emotional impact	Increased anxiety Anxiety seeing others further along in dementia Managing aggression not explored Increased stress due to the additional time required for digital solutions
	Service issues	Unclear service scope Confusing who the point of contact is Limited discussion of progress and planning for future needs Negative experiences of communication with staff Staff nervousness and insensitivity during assessments Limited duration of peer support Interventions coming too late
	Activity relevance	Limited activities relevant to interests and hobbies Peer support system logistics being difficult Digital solutions less relevant for early stages of dementia
	Social dynamics	Success dependent on PLWD/carer relationship Pre‐existing family dynamics affecting commitment
	Logistical challenges	Onus on carers to locate and facilitate support Some carers had to give up employment or travel far distances to ensure PLWD attendance Carers are sometimes overwhelmed by information overload
	Outcomes	PLWD enjoying activities but not recalling them later Limited support for emotional management and planning for future needs Limited effectiveness of interventions for emotions and future planning

For carers of PLWD, the outcomes from SP interventions demonstrate numerous benefits. These include: *enhanced independence*, indicated by a reduced caring load and respite from caregiving duties, coupled with empowerment through knowledge [7, 24, 29, 31, 34, 39, 43, 45, 46, 48, 51, 52, 55, 58, 62, 68]; *improved mood and well‐being*, including reduced stress and anxiety and improved emotional responses [11, 24, 31, 33, 37, 38, 40, 44, 45, 46, 47, 51, 55, 58, 62, 63]; *increased social connectedness*, such as socialisation, peer support and a sense of belonging [5, 26, 29, 33, 34, 35, 36, 39, 40, 42, 43, 45, 55]; *mental and cognitive benefits*, including better understanding of dementia, improved coping skills and normalisation of the dementia experience [11, 42, 44, 46, 55, 56, 63]; *empowerment and identity*, where carers retain their sense of purpose and feel valued [7, 11, 29, 30, 34, 43, 44, 46]; *practical support and resources*, highlighting better decision‐making, assistance with financial and legal matters, and support from health services [7, 11, 25, 27, 30, 33, 41, 44, 46, 49, 51, 53, 54, 55, 56, 57, 62]; *quality of life improvements*, including enhanced well‐being, resilience and confidence in caregiving capabilities [5, 23, 30, 33, 57]; *positive PLWD–carer relationships*, such as improved interactions with PLWD and reduced conflict [5, 8, 11, 24, 26, 30, 42, 52, 53, 55, 56, 58]; *acceptance and adjustment*, suggesting better coping strategies and facilitated acceptance of the dementia diagnosis [34, 37, 41, 43, 44, 55]; and finally, *increased security and comfort*, indicated by a sense of relief and reduced burden through continuous support [5, 27, 30, 51].

The negative outcomes included *intervention suitability*, where carers of PLWD felt poorly prepared because of a lack of information, or the intervention was not flexible to their support needs [11, 24, 27, 38, 61]; *emotional impact*, where carers experienced increased anxiety, stress from seeing others further along in the dementia journey, and the management of PLWD aggression towards carers not being adequately explored [38, 43, 49, 55, 63, 64]; *service issues*, such as unclear service scope, confusion about who the SP point of contact was, and negative experiences regarding staff communication during appointments [11, 24, 27, 38, 63]; *activity relevance*, such as limited activities matching interests and logistical difficulties with peer support systems [63]; *affected PLWD–carer social dynamics*, whereby the dependency on the relationship with PLWD and pre‐existing family dynamics becomes strained [29, 31, 42]; and *logistical challenges*, where the onus on carers to locate and facilitate support, sometimes leading to employment sacrifices and long travel distances was highlighted [39, 56, 63].

Seven studies reported how outcome measures for carers of PLWD were assessed [32, 33, 34, 40, 47, 60, 64], which indicated varying assessments across several domains. These domains included: *mental and psychological well‐being*, assessed by the Short Warwick–Edinburgh Mental Well‐being Scale (SWEMWBS) [40]; *mood‐related outcomes*, such as depression and anxiety, measured by the General Health Questionnaire 28 item (GHQ‐28), the Positive and Negative Affect Schedule (PANAS), the Caregiver Strain Index (CSI) and the Hospital Anxiety and Depression Scale (HADS) [32, 47, 60, 64]; *quality of life* evaluated with instruments such as the EuroQol‐5 dimensions (EQ‐5D‐5L), the Dementia Quality of Life Proxy Instrument (DEMQoL‐Proxy) and the Adult Social Care Outcomes Toolkit (ASCOT) [32, 33, 34, 64]; *daily activities*, assessed with the Bristol Activities of Daily Living Scale (BADLS) [60]; *self‐management*, including a sense of competence, assessed by the Sense of Competence Questionnaire (SCQ) [60]; and finally, *the quality of the caregiving relationship* measured using the Quality of the Caregiving Relationship (QCPR) [64].

## 4 Discussion

### 4.1 Summary of Main Findings

The literature on SP for carers of PLWD in the United Kingdom is varied. Studies mainly focus on female carers of PLWD over 40, with various relationships between the person living with dementia and the carer. SP interventions vary in frequency and encompass activities that could be delivered exclusively for the carer or via the carer to the PLWD; some studies were unclear on this, making reporting results difficult. Services are often provided collaboratively by the NHS, charities and integrated services, with referrals from diverse sources. Connectors, including clinical, multidisciplinary and non‐clinical staff from community organisations, link carers of PLWD to these interventions. However, the terminology regarding the connectors' roles and remit differs and establishing whether referrals are primarily for the person living with dementia or the carer of PLWD is difficult. Carers of PLWD participate in SP for emotional and practical support, coping strategies, social engagement, dementia education and empowerment. Barriers to participation include cultural insensitivity, practical issues, health problems, psychosocial concerns and communication challenges. Positive outcomes included improved carer mood, social connections, practical support, quality of life and better PLWD–carer relationships. However, intervention suitability, emotional impact, relevance and strained PLWD–carer relationships were associated with negative outcomes. Overall, SP for carers of PLWD hints at favourable outcomes, but its implementation is patchy, with co‐ordination within the pathway lacking a uniform SP model for carers of PLWD that clearly separates their needs from the person living with dementia and addresses the logistical challenges of attending SP interventions.

### 4.2 Comparison With Existing Literature

This review reaffirms the inconsistent nature of SP in terms of scope, stakeholders and procedures reported in other literature in this domain [14, 70, 71]. Results in both *Part 1* (PLWD) [12] and *Part 2* (carers of PLWD) of this two‐part series largely concur that SP is a promising intervention within dementia care but this paper (Part 2) sheds further light on the PLWD–carer relationship and the current SP pathway's lack of consideration that PLWD and carers' holistic needs are likely to be different to one another, or even at odds. Carers of PLWD identified support as a key benefit and reason for participating in SP, but the support was often generalised rather than tailored to specific carer needs. A qualitative study on culturally relevant SP for Pakistani carers also found carer‐specific SP programmes lacked personalisation to the carer's holistic needs and did not consider wider cultural factors or how carers often go unnoticed and slip through the SP net due to a lack of carer self‐identification [72]. This review highlights similar challenges for carers of PLWD.

The findings reflect the complexity of how and when carers of PLWD seek help, demonstrated in this review by a variety of referral routes and stakeholders within the pathway. This complexity is potentially exacerbated by the ‘hidden’ nature of caregiving and the broader social, cultural and economic influences on individuals' health [73, 74]. However, integrating such complexity within an SP model is challenging, suggesting the need for a structured model for carers of PLWD that encompasses clearly defined core components that are flexible to the inevitable contextual variables that are innate to caregiving.

### 4.3 Strengths and Limitations

This systematic review showcases a variety of innovative approaches offering valuable insights for policymakers and practitioners. However, it is limited by the included studies' heterogeneity, making comparison of outcomes challenging and conclusions difficult to draw. Furthermore, evidence of effectiveness was difficult to determine due to gaps in the literature, findings were difficult to generalise due to data heterogeneity, and potential biases in study selection and publication necessitate cautious interpretation, thus highlighting the need for further primary research. Another limitation of this review is that, due to its explicit focus on SP interventions for carers of PLWD, it does not include evidence on interventions aimed specifically at PLWD–carer dyads. Although initially intended to explore the dyadic approach, the complexity and extensive nature of the available evidence necessitated splitting the review into separate manuscripts targeting carers and PLWD individually. As a consequence, valuable insights concerning the feasibility, challenges and implementation complexities associated with dyadic interventions have not been captured here and merit exploration in dedicated future research.

### 4.4 Implications for Policy and Practice

This review identifies components of SP interventions that could be integrated into national dementia care and carer‐related strategy and policy. It provides guidance on resource allocation and demonstrates the positive and negative health outcomes associated with SP for carers of PLWD. The evidence from this review supports policies that encourage person‐centred, holistic approaches and greater collaboration between SP stakeholders to ensure coordinated, timely and carer‐focused support.

This review provides insights into ways to improve SP service delivery by identifying reasons carers of PLWD do and do not participate in SP intervention, which can help clinical and community practitioners design tailored SP interventions in the hope of improving SP uptake and effectiveness for carers of PLWD.

## 5 Future Research

Future research to evaluate the long‐term effects of SP on PLWD carers is necessary, including developing appropriate metrics to measure effectiveness. Currently, the absence of standardised assessments and robust methodological designs limits the clarity around long‐term impacts; developing an evaluation framework informed by mixed‐methods studies and randomised controlled trials (RCTs) included in this systematic review could provide valuable insights. Some studies highlighted evidence of researchers enabling the SP pathway; future studies need to be conscious of this so SP can be studied more objectively. Recognising and mitigating researcher influence will help ensure that outcomes accurately represent the effectiveness of SP. Furthermore, the core components of SP require more exploration to understand their mechanisms of action, change, impact on carers of PLWD and whether they are cost‐effective, as currently this evidence is limited. A clearer understanding of these mechanisms and cost implications can better guide policymakers and healthcare providers towards investing in effective SP interventions. Finally, the PLWD–carer relationship and its effects on SP uptake and maintenance require more detailed exploration. Detailed insights into the dynamics of this relationship will improve targeted intervention strategies, thus enhancing uptake and long‐term maintenance of SP among carers. Additionally, integrating scales and assessment tools identified from existing robust studies into screening or intake processes could help systematically direct carers to the most appropriate services and interventions.

## 6 Conclusion

This review provides a foundation for a comprehensive understanding of SP interventions for carers of PLWD. However, SP in this context is complex, with participation often being opportunistic and initiated by various stakeholders and institutions that operate in a largely uncoordinated process. Whilst evidence suggests that SP is a promising intervention for carers of PLWD, its long‐term impacts and specific mechanisms of action remain unclear and unexplored.

## Author Contributions

**Jessica Marshall:** conceptualisation, methodology, software, data curation, investigation, formal analysis, project administration, visualisation, writing – original draft, writing – review and editing. **Evie Papavasiliou:** conceptualisation, methodology, software, data curation, investigation, formal analysis, project administration, visualisation, writing – original draft, writing – review and editing, validation. **Louise Allan:** writing – review and editing, validation. **Katherine Bradbury:** writing – review and editing, validation. **Chris Fox:** conceptualisation, funding acquisition, writing – review and editing, validation. **Matthew Hawkes:** methodology, writing – review and editing, data curation. **Anne Irvine:** writing – review and editing, validation. **Esme Moniz‐Cook:** writing – review and editing, validation. **Aimee Pick:** writing – review and editing. **Marie Polley:** writing – review and editing, validation. **Amy Rathbone:** writing – review and editing. **Joanne Reeve:** writing – review and editing, validation. **Dame Louise Robinson:** writing – review and editing, validation. **George Rook:** writing – review and editing, validation. **Euan Sadler:** writing – review and editing, validation. **Emma Wolverson:** writing – review and editing, validation. **Sarah Walker:** conceptualisation, methodology, software, data curation, investigation, validation, formal analysis, visualisation, project administration, writing – original draft, writing – review and editing. **Jane Cross:** conceptualisation, methodology, investigation, validation, writing – review and editing, writing – original draft, funding acquisition, visualisation, supervision, project administration, data curation.

## The SPLENDID Collaboration

Prof. Chris Fox, Dr Jane Cross, Prof. Louise Allan, Prof. Anthony Avery, Dr Katherine Bradbury, Anne Irvine, Jessica Marshall, Prof. Antonieta Medina‐Lara, Prof. Esme Moniz‐Cook, Nia Morrish, Prof. Martin Orrell, Dr Evie Papavasiliou, Aimee Pick, Prof. Fiona Poland, Dr Marie Polley, Dr Amy Rathbone, Prof. Joanne Reeve, Prof. Dame Louise Robinson, George Rook, Dr Euan Sadler, Dr Kritika Samsi, Prof. Lee Shepstone, Dr Sarah Walker and Dr Emma Wolverson are members of the SPLENDID Collaboration.

## Disclosure

All International Committee of Medical Journal Editors (ICMJE) conflict of interest forms are completed by each author and located in File S2 in Supporting Informatiom.

## Conflicts of Interest

Euan Sadler declares that they are a NIHR Research for Patient Benefit (RfPB) funding committee panel member.

Joanne Reeve declares they are involved in the NIHR HSDR 130247 grant named: Understanding the Implementation of Link Workers in Primary Care: A Realist Evaluation to Inform Current and Future Policy.

Louise Robinson declares they received payment/honoraria for educational resource production and lectures from Lilly UK.

Katherine Bradbury declares that they are a co‐applicant on this grant, and payments were made to the University of Southampton to pay for her time (10% full‐time equivalent salary).

## Supporting information

Figure S1: Process Oriented Logic model (iteration 3).

File S1 Sample Search String.

File S2: Declarations of Conflicting Interests.

Table S1: PICOTS Inclusion/Exclusion Criteria.

## Data Availability

The authors have nothing to report.

## References

[1] World Health Organization , “World failing to Address Dementia Challenge,” Accessed November 20, 2024, https://www.who.int/news/item/02-09-2021-world-failing-to-address-dementia-challenge.

[2] A. Hansen , S. Hauge , and Å. Bergland , “Meeting Psychosocial Needs for Persons With Dementia in Home Care Services—a Qualitative Study of Different Perceptions and Practices Among Health Care Providers,” BMC Geriatrics 17 (2017): 211, 10.1186/s12877-017-0612-3. 28893181 PMC5594550

[3] Q. Fan , L. DuBose , M. G. Ory , et al., “Financial, Legal, and Functional Challenges of Providing Care for People Living With Dementia and Needs for a Digital Platform: Interview Study Among Family Caregivers,” JMIR Aging 6 (2023): e47577, 10.2196/47577. 37526513 PMC10509746

[4] C. Bamford , J. Wilcock , G. Brunskill , et al., “Improving Primary Care‐Based Post‐Diagnostic Support for People Living With Dementia and Carers: Developing a Complex Intervention Using the Theory of Change,” PLoS One 18 (2023): e0283818, 10.1371/journal.pone.0283818. 37134099 PMC10155958

[5] D. Palmer , J. Wheeler , E. Hendrix , P. N. Sango , and E. Hatzidimitriadou , Social Prescribing in Bexley: Pilot Evaluation Report (Canterbury Christ Church University, 2017).

[6] J. Gallacher and A. Burns , “Social Prescribing for Dementia,” Lancet Neurology 20 (2021): 707–708.

[7] C. Femiola and M. Tilki , “Dementia Peer Support: Service Delivery for the People, by the People,” Working With Older People 21 (2017): 243–250, 10.1108/WWOP-08-2017-0020.

[8] C. Dayson , C. Harris , and A. Woodward , “Evaluation of Age Better in Sheffield: Qualitative Insights Into Interventions to Address Social Isolation and Loneliness,” Centre for Regional Economic and Social Research, accessed December 4, 2024, https://www.shu.ac.uk/centre-regional-economic-social-research/publications/evaluation-of-age-better-in-sheffield-qualitative-insights-into-interventions-to-address-social.

[9] M. Bertotti , C. Frostick , P. Hutt , R. Sohanpal , and D. Carnes , “A Realist Evaluation of Social Prescribing: An Exploration Into the Context and Mechanisms Underpinning a Pathway Linking Primary Care With the Voluntary Sector,” Primary Health Care Research & Development 19 (2018): 232–245, 10.1017/S1463423617000706. 29215328 PMC5904290

[10] J. V. Pescheny , Y. Pappas , and G. Randhawa , “Facilitators and Barriers of Implementing and Delivering Social Prescribing Services: A Systematic Review,” BMC Health Services Research 18 (2018): 86, 10.1186/s12913-018-2893-4. 29415720 PMC5803993

[11] C. Bamford , A. Wheatley , G. Brunskill , et al., “Key Components of Post‐Diagnostic Support for People With Dementia and Their Carers: A Qualitative Study,” PLoS One 16 (2021): e0260506, 10.1371/journal.pone.0260506. 34928972 PMC8687564

[12] PLWD Paper (unpublished manuscript).

[13] J. Marshall , E. Papavasiliou , C. Fox , et al., “Social Prescribing for People Living With Dementia (PLWD) and Their Carers: What Works, for Whom, Under What Circumstances and Why—Protocol for a Complex Intervention Systematic Review,” BMJ Open 14 (2024): e080551, 10.1136/bmjopen-2023-080551. PMC11015224 38589260

[14] K. Husk , K. Blockley , R. Lovell , et al., “What Approaches to Social Prescribing Work, for Whom, and in What Circumstances? A Realist Review,” Health & Social Care in the Community 28 (2020): 309–324, 10.1111/hsc.12839. 31502314 PMC7027770

[15] M. Ouzzani , H. Hammady , Z. Fedorowicz , and A. Elmagarmid , “Rayyan—a Web and Mobile App for Systematic Reviews,” Systematic Reviews 5 (2016): 210, 10.1186/s13643-016-0384-4. 27919275 PMC5139140

[16] D. Gough , “Weight of Evidence: A Framework for the Appraisal of the Quality and Relevance of Evidence,” Research Papers in Education 22 (2007): 213–228, 10.1080/02671520701296189.

[17] J. Popay , H. Roberts , A. Sowden , et al., Guidance on the Conduct of Narrative Synthesis in Systematic Reviews: A Product From the ESRC Methods Programme, 2006, 10.13140/2.1.1018.4643.

[18] J.‐M. Guise , M. E. Butler , C. Chang , M. Viswanathan , T. Pigott , and P. Tugwell , “AHRQ Series on Complex Intervention Systematic Reviews—Paper 6: PRISMA‐CI Extension Statement and Checklist,” Journal of Clinical Epidemiology 90 (2017): 43–50, 10.1016/j.jclinepi.2017.06.016. 28720516

[19] J.‐M. Guise , M. Butler , C. Chang , M. Viswanathan , T. Pigott , and P. Tugwell , “AHRQ Series on Complex Intervention Systematic Reviews—Paper 7: PRISMA‐CI Elaboration and Explanation,” Journal of Clinical Epidemiology 90 (2017): 51–58, 10.1016/j.jclinepi.2017.06.017. 28720513

[20] L. M. Anderson , M. Petticrew , E. Rehfuess , et al., “Using Logic Models to Capture Complexity in Systematic Reviews: Logic Models in Systematic Reviews,” Research Synthesis Methods 2 (2011): 33–42, 10.1002/jrsm.32. 26061598

[21] E. A. Rehfuess , A. Booth , L. Brereton , et al., “Towards a Taxonomy of Logic Models in Systematic Reviews and Health Technology Assessments: A Priori, Staged, and Iterative Approaches,” Research Synthesis Methods 9 (2018): 13–24, 10.1002/jrsm.1254. 28677339

[22] S. Ahmed , J. Hughes , S. Davies , et al., “Specialist Services in Early Diagnosis and Support for Older People With Dementia in England: Staff Roles and Service Mix,” International Journal of Geriatric Psychiatry 33 (2018): 1280–1285, 10.1002/gps.4925. 29932255

[23] F. Akhtar , N. Greenwood , R. Smith , and A. Richardson , “Dementia Cafés: Recommendations From Interviews With Informal Carers,” Working With Older People 21 (2017): 236–242, 10.1108/WWOP-07-2017-0018.

[24] H. Al‐Janabi , C. McLoughlin , J. Oyebode , N. Efstathiou , and M. Calvert , “Six Mechanisms Behind Carer Wellbeing Effects: A Qualitative Study of Healthcare Delivery,” Social Science & Medicine 235 (2019): 112382, 10.1016/j.socscimed.2019.112382. 31326132

[25] M. Atcha , Access to Dementia Diagnosis and Support in a Diverse South Asian Community: A Qualitative Study (Lancaster University, 2018).

[26] K. Baker and A. Irving , “Co‐Producing Approaches to the Management of Dementia Through Social Prescribing,” Social Policy & Administration 50 (2016): 379–397, 10.1111/spol.12127.

[27] C. Bamford , M. Poole , K. Brittain , et al., “Understanding the Challenges to Implementing Case Management for People With Dementia in Primary Care in England: A Qualitative Study Using Normalization Process Theory,” BMC Health Services Research 14 (2014): 549, 10.1186/s12913-014-0549-6. 25409598 PMC4232624

[28] N. Brookes , “Implementation of a Community‐Based Approach to Dementia Care in England: Understanding the Experiences of Staff,” Journal of Social Service Research 43 (2017): 336–345, 10.1080/01488376.2016.1242448.

[29] A. Brooks , L. Farquharson , K. Burnell , and G. Charlesworth , “A Narrative Enquiry of Experienced Family Carers of People With Dementia Volunteering in a Carer Supporter Programme,” Journal of Community & Applied Social Psychology 24 (2014): 491–502, 10.1002/casp.2188.

[30] L. J. E. Brown , Z. Aldridge , A. Pepper , I. Leroi , and K. H. Dening , “‘It's Just Incredible the Difference It Has Made’: Family Carers' Experiences of a Specialist Lewy Body Dementia Admiral Nurse Service,” Age and Ageing 51 (2022): 1–5, 10.1093/ageing/afac207. PMC9548332 36209508

[31] J. Burgess , J. Wenborn , L. Di Bona , M. Orrell , and F. Poland , “Taking Part in the Community Occupational Therapy in Dementia UK Intervention From the Perspective of People With Dementia, Family Carers and Occupational Therapists: A Qualitative Study,” Dementia 20 (2021): 2057–2076, 10.1177/1471301220981240. 33371738

[32] G. Charlesworth , L. Shepstone , E. Wilson , M. Thalanany , M. Mugford , and F. Poland , “Does Befriending by Trained Lay Workers Improve Psychological Well‐Being and Quality of Life for Carers of People With Dementia, and at What Cost? A Randomised Controlled Trial,” Health Technology Assessment 12, no. 4 (2008): 1–78, 10.3310/hta12040. 18284895

[33] C. L. Clarke , S. E. Keyes , H. Wilkinson , et al., HEALTHBRIDGE: The National Evaluation of Peer Support Networks and Dementia Advisers (Department of Health Policy Research Programme Project, 2013).

[34] C. L. Clarke , S. E. Keyes , H. Wilkinson , et al., “‘I Just Want to Get on With My Life’: A Mixed‐Methods Study of Active Management of Quality of Life in Living With Dementia,” Ageing and Society 38 (2018): 378–402, 10.1017/S0144686X16001069.

[35] V. Egdell , “Development of Support Networks in Informal Dementia Care: Guided, Organic, and Chance Routes Through Support,” Canadian Journal on Aging/La Revue Canadienne du Vieillissement 31 (2012): 445–455, 10.1017/S0714980812000323. 23021103

[36] V. Egdell , “The Needs of Informal Carers for People With Dementia,” British Journal of Healthcare Management 18 (2012): 628–635, 10.12968/bjhc.2012.18.12.628.

[37] B. Field , E. Coates , and G. Mountain , “Influences on Uptake of a Community Occupational Therapy Intervention for People With Dementia and Their Family Carers,” British Journal of Occupational Therapy 82 (2019): 38–47, 10.1177/03080226188044.

[38] B. Field , E. Coates , and G. Mountain , “What Influences Uptake of Psychosocial Interventions by People Living With Early Dementia? A Qualitative Study,” Dementia 20 (2021): 2668–2688, 10.1177/14713012211007397. 33956547 PMC8723173

[39] C. Giebel , K. Hanna , H. Tetlow , et al., “‘A Piece of Paper Is Not the Same as Having Someone to Talk to’: Accessing Post‐Diagnostic Dementia Care Before and Since COVID‐19 and Associated Inequalities,” International Journal for Equity in Health 20 (2021): 76, 10.1186/s12939-021-01418-1. 33706774 PMC7948657

[40] C. Giebel , N. Morley , and A. Komuravelli , “A Socially Prescribed Community Service for People Living With Dementia and Family Carers and Its Long‐Term Effects on Well‐Being,” Health & Social Care in the Community 29 (2021): 1852–1857, 10.1111/hsc.13297. 33528081

[41] C. Giebel , C. Sutcliffe , F. Darlington‐Pollock , et al., “Health Inequities in the Care Pathways for People Living With Young‐ and Late‐Onset Dementia: From Pre‐COVID‐19 to Early Pandemic,” International Journal of Environmental Research and Public Health 18 (2021): 686, 10.3390/ijerph18020686. 33466948 PMC7831042

[42] S. Górska , K. Forsyth , S. Prior , L. Irvine , and P. Haughey , “Family Group Conferencing in Dementia Care: An Exploration of Opportunities and Challenges,” International Psychogeriatrics 28 (2016): 233–246, 10.1017/S1041610215001507. 26427300

[43] N. Greenwood , R. Smith , F. Akhtar , and A. Richardson , “A Qualitative Study of Carers' Experiences of Dementia Cafés: A Place to Feel Supported and be Yourself,” BMC Geriatrics 17 (2017): 164, 10.1186/s12877-017-0559-4. 28743253 PMC5527402

[44] A. W. Griffiths , E. Shoesmith , C. Sass , P. Nicholson , and D. Charura , “Relational Counselling as a Psychosocial Intervention for Dementia: Qualitative Evidence From People Living With Dementia and Family Members,” Dementia 20 (2021): 2091–2108, 10.1177/1471301220984912. 33382000 PMC8361473

[45] S. Griffiths , L. Weston , S. Morgan‐Trimmer , et al., “Engaging Stakeholders in Realist Programme Theory Building: Insights From the Prospective Phase of a Primary Care Dementia Support Study,” International Journal of Qualitative Methods 21 (2022): 160940692210775, 10.1177/16094069221077.

[46] P. Hewitt , C. Watts , J. Hussey , K. Power , and T. Williams , “Does a Structured Gardening Programme Improve Well‐Being in Young‐Onset Dementia? A Preliminary Study,” British Journal of Occupational Therapy 76 (2013): 355–361, 10.4276/030802213X1375704016827.

[47] S. Hoskins , M. Coleman , and D. McNeely , “Stress in Carers of Individuals With Dementia and Community Mental Health Teams: An Uncontrolled Evaluation Study,” Journal of Advanced Nursing 50 (2005): 325–333, 10.1111/j.1365-2648.2005.03396.x. 15811112

[48] F. Kelly and A. Innes , “Facilitating Independence: The Benefits of a Post‐Diagnostic Support Project for People With Dementia,” Dementia 15 (2016): 162–180, 10.1177/1471301214520780. 24535818

[49] L. O. J. Killin , T. C. Russ , S. K. Surdhar , et al., “Digital Support Platform: A Qualitative Research Study Investigating the Feasibility of an Internet‐Based, Post‐Diagnostic Support Platform for Families Living With Dementia,” BMJ Open 8 (2018): e020281, 10.1136/bmjopen-2017-020281. PMC5898353 29654028

[50] K. A. Levin , S. Lithgow , M. Miller , and J. Carson , “Post‐Diagnostic Support for Dementia: What Can be Learned From Service Providers' Experiences, Model Variation and Information Recording?,” Health Education 118 (2018): 320–338, 10.1108/HE-08-2017-0042.

[51] J. Ling , K. McCabe , A. Crosland , L. Kane , and J. Eberhardt , “Evaluating the Effects of a Multicomponent Support Service for People Recently Diagnosed With Dementia and Their Carers: A Qualitative Study,” Health Expectations 26 (2023): 1628–1635, 10.1111/hex.13767. 37086030 PMC10349222

[52] R. MacRae , E. Macrae , and L. Carlin , “Modifying Walking Football for People Living With Dementia: Lessons for Best Practice,” Sport in Society 25 (2022): 1405–1418, 10.1080/17430437.2020.1825383.

[53] L. Maio , J. Botsford , and S. Iliffe , “Family Carers' Experiences of the Admiral Nursing Service: A Quantitative Analysis of Carer Feedback,” Aging & Mental Health 20 (2016): 669–675, 10.1080/13607863.2015.1052776. 26062969

[54] A. McDonald and B. Heath , “Developing Services for People With Dementia,” Working With Older People 13 (2009): 18–21, 10.1108/13663666200900045.

[55] A. Milne , R. Guss , and A. Russ , “Psycho‐Educational Support for Relatives of People With a Recent Diagnosis of Mild to Moderate Dementia: An Evaluation of a ‘Course for Carers’,” Dementia 13 (2014): 768–787, 10.1177/1471301213485233. 24339082

[56] G. Mountain , J. Wright , C. L. Cooper , et al., “An Intervention to Promote Self‐Management, Independence and Self‐Efficacy in People With Early‐Stage Dementia: The Journeying Through Dementia RCT,” Health Technology Assessment 26 (2022): 1–152, 10.3310/KHHA0861. PMC9376803 35536231

[57] H. Piercy , S. Fowler‐Davis , M. Dunham , and C. Cooper , “Evaluation of an Integrated Service Delivering Post‐Diagnostic Care and Support for People Living With Dementia and Their Families,” Health & Social Care in the Community 26 (2018): 819–828, 10.1111/hsc.12592. 30033620

[58] L. Prendergast , G. Toms , D. Seddon , R. Tudor Edwards , B. Anthony , and C. Jones , “‘It Was Just—Everything Was Normal’: Outcomes for People Living With Dementia, Their Unpaid Carers, and Paid Carers in a Shared Lives Day Support Service,” Aging & Mental Health 27 (2023): 1282–1290, 10.1080/13607863.2022.2098921. 35848206

[59] K. Sprange , J. Beresford‐Dent , G. Mountain , et al., “Journeying Through Dementia Randomised Controlled Trial of a Psychosocial Intervention for People Living With Early Dementia: Embedded Qualitative Study With Participants, Carers and Interventionists,” Clinical Interventions in Aging 16 (2021): 231–244, 10.2147/CIA.S293921. 33574660 PMC7872215

[60] J. Wenborn , A. G. O'Keeffe , G. Mountain , et al., “Community Occupational Therapy for People With Dementia and Family Carers (COTiD‐UK) Versus Treatment as Usual (Valuing Active Life in Dementia [VALID]) Study: A Single‐Blind, Randomised Controlled Trial,” PLoS Medicine 18 (2021): e1003433, 10.1371/journal.pmed.1003433. 33395437 PMC7781374

[61] A. Wheatley , C. Bamford , G. Brunskill , L. Booi , K. H. Dening , and L. Robinson , “Implementing Post‐Diagnostic Support for People Living With Dementia in England: A Qualitative Study of Barriers and Strategies Used to Address These in Practice,” Age and Ageing 50 (2021): 2230–2237, 10.1093/ageing/afab114. 34240114 PMC8675435

[62] N. L. Wheeler , J. L. Allen , P. Bentham , E. Cook , Y. Davies , and P. McDonald , “A Specialist Welfare Advice and Advocacy Service for People With Early Onset Dementia: Nicola Louise Wheeler and Colleagues Outline the Success of a Joint Initiative to Support the Increasing Number of People With This Condition,” Mental Health Practice 18 (2015): 20–26, 10.7748/mhp.18.10.20.e948.

[63] R. Willis , J. Chan , J. Murray , D. Matthews , and S. Banerjee , “People With Dementia and Their Family Carers' Satisfaction With a Memory Service: A Qualitative Evaluation Generating Quality Indicators for Dementia Care,” Journal of Mental Health 18 (2009): 26–37, 10.1080/09638230701529681.

[64] R. Woods , E. Bruce , R. Edwards , et al., “REMCARE: Reminiscence Groups for People With Dementia and Their Family Caregivers—Effectiveness and Cost‐Effectiveness Pragmatic Multicentre Randomised Trial,” Health Technology Assessment 16, no. 48 (2012), 10.3310/hta16480. 23211271

[65] A. Ahmed , M. Wilding , R. Haworth‐Lomax , and S. McCaughan , Promoting Diversity and Inclusiveness in Dementia Services in Salford (University of Salford, 2017).

[66] C. Dayson and N. Bashir , The Social and Economic Impact of the Rotherham Social Prescribing Pilot: Main Evaluation Report (Sheffield Hallam University, 2014).

[67] C. Dayson , N. Bashir , E. Bennett , and E. Sanderson , The Rotherham Social Prescribing Service for People With Long‐Term Health Conditions: Annual Evaluation Report (Sheffield Hallam University, 2016).

[68] C. Goodman , A. Arthur , S. Buckner , et al., National Institute for Health Research Policy Research Programme Project Dementia Friendly Communities: The DEMCOM Evaluation (PR‐R15‐0116‐21003). 2020.

[69] C. Muhl , K. Mulligan , I. Bayoumi , R. Ashcroft , and C. Godfrey , “Establishing Internationally Accepted Conceptual and Operational Definitions of Social Prescribing Through Expert Consensus: A Delphi Study,” BMJ Open 13 (2023): e070184, 10.1136/bmjopen-2022-070184. PMC10351285 37451718

[70] J. Brandling and W. House , Investigation Into the Feasibility of a Social Prescribing Service in Primary Care: A Pilot Project (University of Bath and Bath and Northeast Somerset NHS Primary Care Trust, 2007).

[71] R. Kimberlee , “What Is Social Prescribing?,” Advances in Social Sciences Research Journal 2 (2015): 102–110, 10.14738/assrj.21.808.

[72] S. McMullen , S. Poduval , M. Armstrong , et al., “A Qualitative Exploration of the Role of Culturally Relevant Social Prescribing in Supporting Pakistani Carers Living in the UK,” Health Expectations 27, no. 6 (2024): e70099, 10.1111/hex.70099. 39523708 PMC11551476

[73] C. Barber , “Hidden Caregivers: Providing Appropriate Services,” BJHA 6 (2012): 530–533, 10.12968/bjha.2012.6.11.530.

[74] S. Knowles , R. Combs , S. Kirk , M. Griffiths , N. Patel , and C. Sanders , “Hidden Caring, Hidden Carers? Exploring the Experience of Carers for People With Long‐Term Conditions,” Health & Social Care in the Community 24 (2016): 203–213, 10.1111/hsc.12207. 25706665 PMC4744729

