# Supplementary material for: Reimagining Dementia Care: A Complex Intervention Systematic Review on Optimising Social Prescribing (SP) for Carers of People Living With Dementia (PLWD) in the United Kingdom
Source: Health Expect. 2025 May 10;28(3):e70286. doi: 10.1111/hex.70286 (PMC12064994; doi:10.1111/hex.70286)
Supplement: Supplementary file 1 — Figure S1: Process Oriented Logic model (iteration 3). [file HEX-28-e70286-s004.docx]

**PARTICIPANTS**

**PLWD:** Anyone of any age, sex, ethnicity and socio-economic status living with diagnosed dementia^1^ of any subtype

**Carers of PLWD^2^:** Anyone of any age, sex, ethnicity and socio-economic status who provides unpaid care, help or support to a family member, partner or friend who needs help because they have a dementia diagnosis

**Theory:**

- Aims of social prescribing^3^ for carers of PLWD
- Any implicit or explicit ideas about how social prescribing works for carers of PLWD in what circumstances and why

**Design:**

**Execution:**

- Timing of social prescribing (not constrained by specific timings)
- Level of engagement with social prescribing (unlimited)
- Intensity of social prescribing (varied)
- Duration of social prescribing (varied)
- Process of social prescribing (identifier (optional), connector^4^, co-produced care plan, voluntary or community sector organisation).

**Components:**

- Types of non-medical intervention^5^: educational, social, cultural, arts, advice, physical activity, volunteering, befriending, therapy, peer support, cognitive, case -management, psychosocial, community & wellbeing, occupational & complementary services

**Delivery^6^:**

**Delivery agents:**

- Any trusted individual acting as a connector in the delivery of social prescribing services, who empowers a person through holistic support and a personalised co-produced care plan^7^ to connect them to voluntary or community sector organisations,^8^ delivered desirably by people with lived experience.

**Delivery mechanisms:**

- NHS (primary & secondary care)
- Charities
- Local authorities
- Community interest groups
- Voluntary or community sector organisations
- Open referral system (including family/self-referral)

**Setting:**

- Community based living^9^ in the UK.
- Variable setting influences the intervention design and delivery.

**OUTCOMES**

- Improved physical and/or mental well-being^10^
- Improved quality of life^11^
- Improved mood and well-being
- Mental & cognitive benefits
- Maintaining a sense of identity

**INTERVENTION**

**IMPLEMENTATION**

**Policy:**

NHS Long Term Plan 2019; A Plan for Scotland 2016-17; Prosperity for all 2017; Planning guidance for COVID-19 recovery; Major conditions strategy: case for change and our strategic framework 2023; Universal Personalised Care: Implementing the Comprehensive Model 2019; NHS Five year Forward View 2014; General Practice Forward View 2018

**Funding:**

Allocation of funds & funding bodies for SP projects; Lack of health budget for ageing population; Social care funding crisis.

**Organisation:**

Available facilities and resources dedicated for implementation of SP projects and connectors, Long waiting lists & ongoing strikes of professionals

**Provider:**

Any evidence relating to attitudes, communication skills, beliefs about capabilities and self-efficacy, emotions, motivation, attitudes towards intervention (multidisciplinary working; lack of commitment), knowledge, skills (lack of skills), training, and professional development of delivery agents (lack of engagement of GPs; lack of training)

**CONTEXT**

**Epidemiological:**

- Ageing population
- Increased number of PLWD
- Timing of dementia diagnoses, type and stage
- Co-morbidities

**Socio-cultural:**

- Dementia stigma, cultural, religious, and language barriers within SP projects that prevent these schemes from being diversity friendly
- Lack of healthcare trust and knowledge amongst ethnic minorities

**Geographical:**

- Infrastructure at a given location (diverse)
- Access (transport, accessibility, and timing of sessions)

**Legal:**

- Mental Capacity Act 2005
- Lasting Power of Attorney (LPA)
- Advance Statement
- Advance Decision

**Socio-economic:**

- Increasing cost/socioeconomic burden for PLWD
- Financial constraints
- Digital Divide

**Figure S1:** Process Oriented Logic model (iteration 3)

**Health**

- Improved capacity for daily living (through practical support and resources)
- Improved social connectedness
- Increased security and comfort by reducing carer burden
- Activities evoking frustration or anxiety
- Pre-existing family dynamics affecting commitment
- Empowered individuals (having a greater say in their lives and health)
- Improved / deteriorated PLWD-carer relationship and communication
- Improved acceptance and adjustment to dementia diagnosis
- Highlighted the onus on PLWD carers to overcome the logistical challenges of SP

**Non -Health**

GLOSSARY OF TERMS & DEFINITIONS*:

1. *Dementia: a diagnosed syndrome related to cognitive and behavioural decline which over time affects memory, problem solving, language, mood, motivation, and behaviour to such an extent that it interferes with a person's daily life and activities.*
2. *Carers of PLWD: Anyone of any age who provides unpaid care, help or support to a family member, partner or friend who needs help because they have a dementia diagnosis.*
3. *Social Prescribing: a means for trusted individuals in clinical and community settings to identify that a person has non-medical, health related social needs and to subsequently connect them to non-clinical supports and services within the community by co-producing a social prescription – a non-medical prescription, to improve health and wellbeing and to strengthen community connections [11 p.9].*
4. *Connector: A trusted individual in a clinical or community setting involved in the delivery of social prescribing services, who empowers a person through holistic support and a personalised co-produced care plan to connect them to voluntary and community sector organisations.*
5. *Non-medical intervention: A non-clinical approach, activity, opportunity, or support scheme.*
6. *Delivery: Intervention delivery describes the ‘how’ (delivery mechanisms), ‘who’ (delivery agents), ‘where’ (setting) of the intervention [25].*
7. *Personalised co-produced care plan: A verbal or written holistic, person-centred adaptive plan based on ‘what matters’ to a person. It is produced in equal partnership with a trusted individual in a clinical or community setting to address non-medical health related needs.*
8. *Voluntary and community sector organisations: A not for profit, non-clinical community asset or scheme that offers people in-person or remote services, activities, opportunities, or support.*
9. *Community based living: Living primarily in the community, either independently or supported by others, including in a residential care home, but not in a nursing home, a hospice or receiving inpatient or end of life care.*
10. *Wellbeing: Exists in two dimensions. Subjective wellbeing relates to how people feel and how they function on a personal level in relations to their financial, health, social, personal, and local environment. Objective wellbeing relates to objective measures of an individual’s being and assumptions about basic human needs and rights.*
11. *Quality of life: A multidimensional measure of an individual’s health in terms of (but not limited to) their physical, psychological, social, personal, and environmental state.*

*Working definitions of terms for which inconsistency and/or ambiguity persists in the current literature were specifically developed drawing on stakeholder conversations, clinical, peer reviewed and grey literature, being adapted to fit the aims of this CISR.

**Green text** = Adaptions, modifications, and additions to the initial logic model (iteration 1). These represent new evidence-based insights, components, and scope that have emerged during the review’s extraction and synthesis stage and SPLENDID CISR PPI Consultations (Round 2).

**Blue text** = Adaptions, modifications, and additions to the initial logic model (iteration 1). These represent new evidence-based insights, components, and scope that have emerged during the review’s writing up and pre-dissemination stage, and the SPLENDID CISR PPI Consultations (Round 3).

NHS Long Term Plan 2019: <https://www.longtermplan.nhs.uk/wp-content/uploads/2019/08/nhs-long-term-plan-version-1.2.pdf>

A Plan for Scotland 2016-2017: <https://www.gov.scot/binaries/content/documents/govscot/publications/strategy-plan/2016/09/plan-scotland-scottish-governments-programme-scotland-2016-17/documents/00505210-pdf/00505210-pdf/govscot%3Adocument/00505210.pdf>

Prosperity for All 2017: <https://www.gov.wales/sites/default/files/publications/2019-02/prosperity-for-all-economic-action-plan.pdf>

Planning Guidance for Covid-19 Recovery: <https://assets.publishing.service.gov.uk/media/5ebbc6e1d3bf7f5d364bfbcf/Our_plan_to_rebuild_The_UK_Government_s_COVID-19_recovery_strategy.pdf>

Major Conditions Strategy: case for change and our strategic framework 2023: <https://www.gov.uk/government/publications/major-conditions-strategy-case-for-change-and-our-strategic-framework/major-conditions-strategy-case-for-change-and-our-strategic-framework--2>

Universal Personalised Care: Implementing the Comprehensive Model 2019: <https://www.england.nhs.uk/wp-content/uploads/2019/01/universal-personalised-care.pdf>

NHS Five Year Forward View 2014: <https://www.england.nhs.uk/wp-content/uploads/2014/10/5yfv-web.pdf>

General Practice Forward View 2018: https://www.rcgp.org.uk/getmedia/44b1c8e8-fa06-470b-b8ae-af91ce58ef96/RCGP-annual-assessment-GP-forward-view-year2-aug-2018.PDF
