# Supplementary material for: Reimagining Dementia Care: A Complex Intervention Systematic Review on Optimising Social Prescribing (SP) for Carers of People Living With Dementia (PLWD) in the United Kingdom
Source: Health Expect. 2025 May 10;28(3):e70286. doi: 10.1111/hex.70286 (PMC12064994; doi:10.1111/hex.70286)
Supplement: Supplementary file 2 — File S1 Sample Search String. [file HEX-28-e70286-s003.docx]

**File S1: Sample Search String**

Database: Medline (Ovid)

1 exp Dementia/

2 dement*.tw.

3 alzheimer*.tw.

4 huntington.tw.

5 lewy body.tw.

6 (cognitive adj1 (decline or dysfunction or disorder*)).tw.

7 (memory adj1 (problem* or loss)).tw.

8 Creutzfeldt-Jakob disease.tw.

9 pick disease.tw.

10 or/1-9

11 ((social or community or psychosocial) adj2 (health* or medicine or consultation or prescri* or activ* or refer* or therap* or program* or interven* or care or based or project*)).tw.

12 (referral adj1 (scheme* or art or befriending or non-clinical or non-med* or information or supported or well-being or wellbeing or guided)).tw.

13 (link* adj1 (scheme* or worker* or community)).tw.

14 linkworker.tw.

15 (Navigator adj1 (patient or care or health* or community or outreach or system* or resource or peer)).tw.

16 exercise.tw.

17 art.tw. /freq=2

18 (sport* adj2 (therap* or program* or interven*)).tw.

19 swim*.tw. /freq=2

20 befriend*.tw.

21 music.tw. /freq=2

22 singing.tw.

23 nature.tw. /freq=2

24 aquatherapy.tw.

25 ecotherapy.tw.

26 gardening.tw.

27 cooking.tw. /freq=2

28 ((sensory adj2 (experience or intervention or group)) or multisensory).tw.

29 ((animal* or pet* or dog* or cat* or pony or ponies) adj2 therap*).tw.

30 conservation.tw.

31 walking.tw.

32 outdoor.tw.

33 horticultural.tw.

34 yoga.tw.

35 mindful*.tw.

36 meditat*.tw.

37 museum.tw.

38 (health advis?r* or medical advis?r* or health trainer* or wellbeing coordinator* or well-being co-ordinator*).tw.

39 (non-drug or non-pharma*).tw.

40 exp Social Support/

41 Social Interaction/

42 (home adj2 prescri*).tw.

43 healthy living.tw.

44 wellness.tw.

45 facilitator*.tw.

46 or/11-45

47 exp United Kingdom/

48 (UK or United Kingdom or England or Ireland or Irish or Scotland or Scottish or Wales or Welsh or Britain or British or NHS or national health service).tw,au,so,gc,in.

49 or/47-48

50 and/10,46,49

51 social prescri*.tw.

52 and/10,51

53 or/50,52

54 limit 53 to yr="2003 -Current"

55 exp Animals/

56 exp Humans/

57 55 not 56

58 54 not 57
