# Supplementary material for: Reimagining Dementia Care: A Complex Intervention Systematic Review on Optimising Social Prescribing (SP) for Carers of People Living With Dementia (PLWD) in the United Kingdom
Source: Health Expect. 2025 May 10;28(3):e70286. doi: 10.1111/hex.70286 (PMC12064994; doi:10.1111/hex.70286)
Supplement: Supplementary file 4 — Table S1: PICOTS Inclusion/Exclusion Criteria. [file HEX-28-e70286-s002.docx]

**Table S1**: PICOTS Inclusion/Exclusion Criteria

| **PICOTS** | **Inclusion** | **Exclusion** |
| --- | --- | --- |
| **Population** | *Person living with dementia:*   - Anyone of any age, sex, ethnicity, and socio-economic status living with diagnosed dementia (of any subtype or stage). - Living in the community (residential care homes included), either independently or supported by others.   *Carers:*   - Of any age, sex, ethnicity, and socio-economic status.   Providing unpaid care help or support to a family member, partner or friend living with diagnosed dementia (of any subtype or stage) but can be receiving a carer’s allowance. | *Person living with dementia:*   - Not having a formal dementia diagnosis. - Living in a nursing home, a hospice or receiving inpatient or end of life care.   *Carers:*   - Anyone providing care to PLWD not having a formal dementia diagnosis.   Anyone providing paid care, help or support to PLWD (of any subtype or stage). |
| **Intervention** | *Social prescribing (defined as):* “a means for trusted individuals in clinical and community settings to identify that a person has non-medical, health related social needs and to subsequently connect them to non-clinical supports and services within the community by co-producing a social prescription – a non-medical prescription, to improve health and wellbeing and to strengthen community connections. [11 p.9]”  **Any non-medical interventions** (activities, groups, or services within the community that meet the practical, social, and emotional needs that affect health and wellbeing of patients living with dementia and/or their carers).  run by  **Any voluntary or community sector organisations** (either community based or virtual)  accessed through  **Any referral route** (such as primary care (GPs), charity, community interest group, local authority, self-referral, carer-referral but not limited to).  via a  **Connector** (a trusted individual in a clinical or community setting involved in the delivery of social prescribing services)  through a  **Personalised co-produced care plan** (a verbal or written holistic, person-centered adaptive plan to address non-medical health related needs based on ‘what matters’ to a person) | - Any medical interventions (e.g., pharmacological). - Any non-medical interventions accessed without a connector being involved and no personalised co-produced care plan used. |
| **Context** | *Published output:*   - Focusing on UK healthcare - Papers of any empirical design - Grey literature (reports)   January 2003-October 2023 | *Published output:*   - Focusing on International healthcare - Reviews - Case studies - Editorials - News   Conference abstracts & proceedings |
| **Outcomes** | Any quantitative (e.g., scores of psychometric instruments and/or other standardised measures) or qualitative (perceptions, thoughts, experiences) individual (health and non-health) and systemic outcomes, including proxy perspectives (see Figure 1). | No restriction |
| **Timing** | No time restriction | No time restriction |
| **Setting** | Community based living anywhere in the UK. | PLWD living in a nursing home, a hospice or receiving inpatient or end of life care. |
